# Integrating Virtual Surgical Planning and 3D-Printed Tools with Iliac Bone Grafts for Orbital and Zygomatic Reconstruction in Hemifacial Microsomia Patients

**DOI:** 10.3390/jcm12247538

**Published:** 2023-12-06

**Authors:** Zhiyang Zhao, Jiahao Bao, Guofang Shen, Ming Cai, Hongbo Yu

**Affiliations:** Department of Oral and Craniomaxillofacial Surgery, Shanghai Ninth People’s Hospital, College of Stomatology, Shanghai Jiao Tong University School of Medicine, National Clinical Research Center for Oral Diseases, Shanghai Key Laboratory of Stomatology, Shanghai Research Institute of Stomatology, No. 639, Zhizaoju Road, Shanghai 200011, China; zzy19980205@sjtu.edu.cn (Z.Z.); baojh0123@sjtu.edu.cn (J.B.); shengf1428@sjtu.edu.cn (G.S.)

**Keywords:** hemifacial microsomia, orthognathic surgery, 3D printing, customized surgical adjuncts, iliac cancellous bone graft

## Abstract

Hemifacial Microsomia (HFM) is the second most common congenital craniofacial malformation syndrome, and the complexity of HFM makes its treatment challenging. The present study aimed to introduce a new approach of utilization of virtual surgical planning (VSP) and 3D-printed surgical adjuncts for maxillofacial reconstruction. Five HFM patients were included in this study. All participants were provided with a full VSP, including the design of osteotomy lines, the design and fabrication of 3D-printed cutting guides, fixation plates, and titanium mesh for implantation. With the assistance of 3D-printed cutting guides and fixation plates, the orbital deformities were corrected, and a 3D-printed titanium mesh combined with iliac cancellous bone graft was applied to reconstruct the zygomatic arch. The surgical accuracy, effectiveness, and bone absorption rate were evaluated. All patients completed the entirely digital treatment process without experiencing severe complications. The surgical adjuncts were effective in aligning the movement of the bone segments with the surgical plan, resulting in mean 3D deviations (1.0681 ± 0.15 mm) and maximum 3D deviations (3.1127 ± 0.44 mm). The image fusion results showed that the patients’ postoperative position of the maxilla, zygoma, and orbital rim was consistent with the virtual surgical plan, with only a slight increase in the area of bone grafting. The postoperative measurements showed significant improvement in the asymmetry indices of Er (AI of Er: from 17.91 ± 3.732 to 5.427 ± 1.389 mm, *p* = 0.0001) and FZ (AI of FZ: from 7.581 ± 1.435 to 4.070 ± 1.028 mm, *p* = 0.0009) points. In addition, the observed bone resorption rate at the 6-month follow-up across the five patients was 45.24% ± 3.13%. In conclusion, the application of VSP and 3D-printed surgical adjuncts demonstrates significant value in enhancing the precision and effectiveness of surgical treatments for HFM. A 3D-printed titanium mesh combined with iliac cancellous bone graft can be considered an ideal alternative for the reconstruction of the zygomatic arch.

## 1. Introduction

Hemifacial Microsomia (HFM), alternatively termed as the First and Second Branchial Arch Syndrome, Craniofacial Microsomia, Oculo-Auriculo-Vertebral Spectrum, or Goldenhar Syndrome, is the second most common congenital craniofacial aberration following orofacial clefts, with a prevalence of 1 in 3000 to 5600 live births [1,2]. It is a congenital disorder leading to developmental dysplasia of structures due to the anomalous fusion of the first and second pharyngeal arches during embryogenesis, affecting an array of skeletal and soft structures across the entire midface and lower face within the craniofacial region, including the ears, orbits, zygomaticomaxillary complex, and mandible [3,4]. Patients with HFM mainly manifest with mandibular hypoplasia, facial asymmetry, malocclusion, orbital anomalies, preauricular fistulas, microphthalmia, microtia, and macroglossia, which gravely impact patients’ appearance and mentality [4,5]. The diversity and complexity of HFM make its treatment challenging, even for highly experienced surgeons [6]. Typically, the management of HFM requires a multidisciplinary and multistage approach. Different surgical approaches have been utilized to correct the facial deformity via bone and soft tissue reconstruction, such as mandibular distraction osteogenesis, orthognathic surgery with bone graft, temporomandibular joint reconstruction, fat grafting, and microvascular-free tissue transfer [4,7,8]. However, since the mandible is often and most noticeably impacted in HFM patients, the majority of reported studies have focused on the correction of mandibular asymmetry, with limited research dedicated to the correction of midfacial asymmetry.

HFM patients often exhibit developmental anomalies in their midfacial bony structures as well. It was reported that the maxillary, orbital, zygomatic, and temporal sizes were different in the affected size of HFM patients compared with the unaffected side [9]. For examples, the orbits showed an abnormal size and position [5]. The maxillary, zygomatic, and temporal bones often manifest hypoplasia, exhibiting diminished dimensions and a flattened morphological profile. These alterations culminate in a recessed zygoma and maxilla, engendering conspicuous facial asymmetry when assessed from an anterior perspective. Anomalies pertaining to the zygomatic arch are likewise prevalent, ranging from a diminished anteroposterior length to the complete agenesis of the arch. Therefore, the restoration of maxillary and zygomatic symmetry and improvement of orbital deformities are of paramount significance for establishing facial balance and enhancing the facial aesthetics of HFM patients.

The advent of virtual surgical planning (VSP) and 3D printing technologies has revolutionized the management of HFM, offering enhanced accuracy and predictability, and allowing for tailored patient-specific solutions [10,11,12]. Studies have shown the effectiveness of VSP and 3D-printed surgical guides in improving surgical outcomes [13,14]. 3D-printed surgical adjuncts can provide a precise fit with the bone and can transfer surgical plans to the actual operations. Additionally, autologous iliac cancellous bone is a favored choice for maxillofacial reconstruction due to its plasticity and sufficient volume, while maintaining the iliac crest’s contour [15]. It presents good plasticity and can provide a sufficient quantity of bone while preserving the external contour of the iliac crest. However, there have been no studies found that evaluate the postoperative outcomes of orbital and zygomatic reconstruction in patients with HFM using three-dimensional printed surgical guides and a titanium mesh.

Therefore, our study aims to fill this gap by introducing a novel surgical approach that utilizes VSP and 3D-printed adjuncts. Specifically, this method involves simultaneous orthognathic surgery with the application of 3D-printed cutting guides and fixation plates to correct the orbital deformities, along with the utilization of 3D-printed personalized titanium plates and titanium mesh combined with iliac cancellous bone graft to reconstruct the zygomatic arch. We evaluate the feasibility and surgical accuracy of this method, and the long-term osteogenesis of bone graft is assessed.

## 2. Materials and Methods

This study was performed according to the Declaration of Helsinki and after approval by the ethics committee of Shanghai Ninth People’s Hospital, School of Medicine, Shanghai Jiao Tong University (SH9H-2021-T64-1). Informed consent was obtained from each participant.

### 2.1. Inclusion and Exclusion of Patients

Five adult HFM patients from 2021 to 2023 at the Department of Oral and Craniomaxillofacial Surgery in Shanghai Ninth People’s Hospital were enrolled in this study. The inclusion criteria were as follows: All patients were diagnosed with Type IIb and III according to the Pruzansky–Kaban classification [16], and they presented with orbital deformities classified as O1 (abnormal orbital size) in the “OMENS+” classification [17]. All patients presented with facial deformities, including severe facial asymmetry, mandibular hypoplasia, zygomaticomaxillary complex hypoplasia, orbital anomalies, occlusal cant, and malocclusion. Patients whose deformities were caused by other congenital disorders or acquired diseases and those who have received surgeries including distraction osteogenesis, costochondral rib graft, and soft tissue reconstruction were excluded from our study. All included HFM participants were treated with orthognathic surgery with the assistance of 3D-printed metal osteotomy guides to restore facial symmetry. Specifically, the correction of orbital deformities was performed with 3D-printed cutting guides and fixation plates (Ti_6_Al_4_V), and the reconstruction of zygomatic arch was conducted using 3D-printed titanium mesh (Ti_6_Al_4_V) combined with iliac cancellous bone.

### 2.2. Virtual Surgical Planning

**Figure 1:**
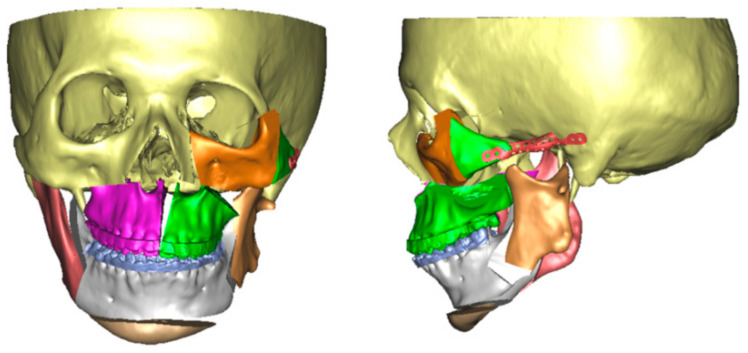
Virtual surgical planning of orthognathic surgery, orbital deformity correction, and zygomatic reconstruction for HFM patients.

**Figure 2:**
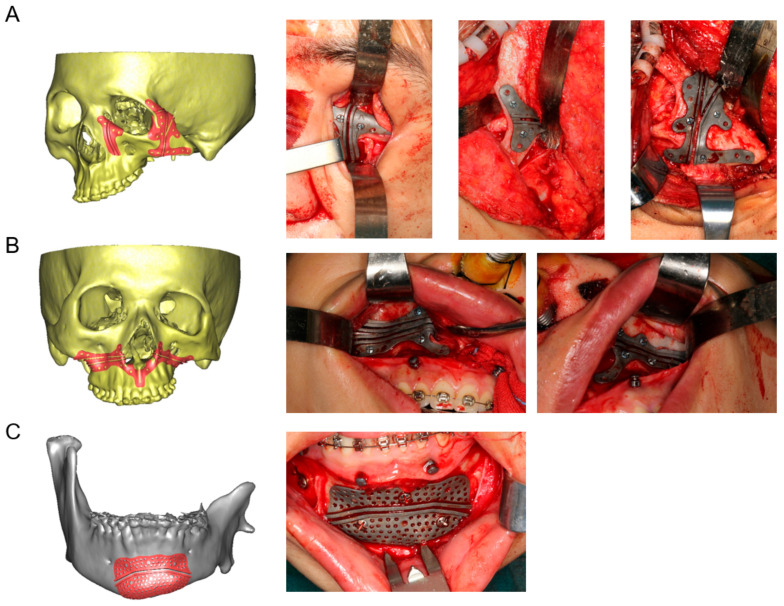
Design and application of titanium cutting guides. (**A**) Design and intraoperative application of 3D-printed titanium cutting guides for orbital deformity correction and zygomatic arch reconstruction. (**B**) Design and intraoperative application of 3D-printed titanium cutting guides for Le Fort I osteotomy. (**C**) Design and intraoperative application of 3D-printed titanium cutting guides for genioplasty.

Patients scheduled for surgery underwent a preoperative evaluation program including history, clinical examination, 2D and 3D photographs, cephalometric and panoramic radiographs, computed tomography (CT) scans, and dental casts. Virtual planning of orthognathic surgery and design of 3D-printed cutting guides, fixation plates, and titanium mesh were performed following the methodology outlined in the previously published article (Figure 1) [13]. In brief, CT scans in Digital Imaging and Communications in Medicine (DICOM) format, acquired from a LightSpeed CT scanner (GE Healthcare, Hatfield, UK), or cone beam CT (CBCT) data from I-CAT imaging system (Imaging Sciences International, Hatfield, PA, USA) were imported into ProPlan CMF 3.0 software (Materialise, Leuven, Belgium) to create the 3D skull model. Then, a composite skull model was established by incorporating CT and digital dental models, and cephalometric landmarks were identified for analysis. Virtual surgical simulations, including Le Fort I osteotomy, bilateral sagittal split ramus osteotomy (BSSRO), genioplasty, correction of orbital deformities, and reconstruction of zygomatic arch, were performed and osteotomy lines were digitally designed via multiplanar (axial, coronal, and sagittal) as well as 3D views following the computer-assisted surgery (CAS) planning procedure (Figure 1 and Figure 2).

### 2.3. Design and Fabrication of 3D-Printed Cutting Guides, Fixation Plates, and Titanium Mesh

After virtual surgical planning and simulation, 3D customized surgical guidance, fixation plates, and mesh were designed employing computer-aided design (CAD)/computer-aided manufacturing (CAM) software—Geomagic Studio 2013 Software (Geomagic, Durham, NC, USA). Files comprising surgical plans including osteotomy lines and the finial planning position of bone segments were imported into Geomagic Studio 2013 software to facilitate the design of customized surgical guides. Then, TiAI64V osteotomy guides were designed for Le Fort I osteotomy, genioplasty, and surgery in periorbital and zygomatic arch region (Figure 2). Moreover, the patient-specific titanium mesh was designed for zygomatic arch reconstruction, and titanium fixation plates were designed for fixation of the osteotomy segments. Subsequently, these guides, mesh, and plates were fabricated using a titanium 3D printer (M2 cusing Mutilaser; CONCEPTLASER, Germany). All titanium products were surface blasted, polished, and cleaned by sonication in acetone, ethanol, and distilled water sequentially for 15 min and then steam autoclaved before surgeries.

### 2.4. Surgical Procedure

**Figure 3:**
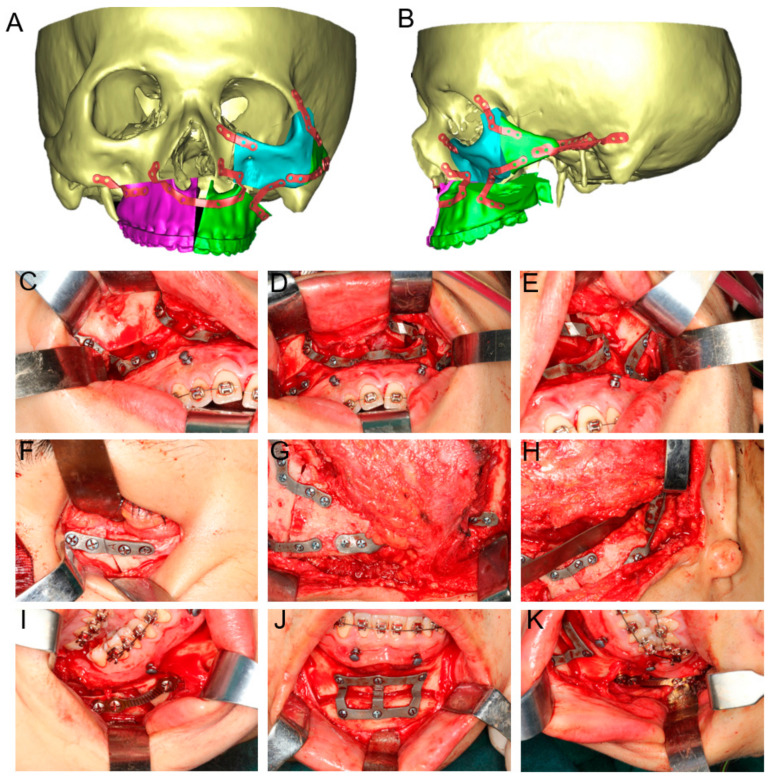
Design and application of titanium fixation plates and titanium mesh. (**A**,**B**) The fixation plates were designed based on the predetermined position of the maxilla and zygoma. (**C**–**E**) Intraoperative application of maxillary 3D-printed personalized fixation titanium plates. (**F**–**H**) The 3D-printed personalized fixed titanium plates and titanium mesh in zygomatic arch reconstruction and orbital deformity correction. (**I**–**K**) Intraoperation application of 3D-printed personalized fixed titanium plates in genioplasty and stock fixation was applied in BSSRO.

In this study, both VSPs and the surgical procedures were performed by the same surgical team. All the surgical procedures were conducted under general anesthesia. First, the maxillary vestibular incision was initiated through mucosa and extended through subcutaneous tissue, muscle, and periosteum. By the subperiosteal dissection, the zygomaticomaxillary complex was exposed. In addition, the maxilla and inferior orbital rim were exposed adequately via a conjunctival incision to create sufficient space for the placement of customized guides and fixation plates. Then, the osteotomy line was methodically marked and defined. Customized 3D-printed osteotomy guides were mounted on the maxilla and zygoma to transfer virtual surgical plans to the actual surgery. With the assistance of cutting guides, a reciprocating saw was utilized to create precise osteotomies. A hemicoronal incision was performed on the affect side of the patient’s scalp, and then, the surgeon proceeded to dissect downward through the subcutaneous tissue to expose the zygomatic bone and the orbit. Zygomatic body, zygomatic process of frontal bone, and zygomatic process of temporal bone were separated according to the plan. During the maxillofacial procedures, the harvesting of cancellous bone from the iliac crest was simultaneously conducted. The surgeon made a precise incision in the skin over the anterior portion of the iliac crest and carefully separated the subcutaneous tissue to expose the anterior superior iliac spine. An osteotomy was then performed on the iliac crest using a bone saw or chisel, and the cancellous bone was meticulously collected from the osteotomized area. Patient-specific 3D-printed titanium plates were employed for internal fixation of zygomatic bone, which can efficiently fixate atypical osteotomies. And the 3D-printed titanium mesh was installed with the predrilled hole method and the zygomatic bone segment was fixed to the temporal bone. The harvested iliac cancellous bone was subsequently filled into this titanium mesh, ensuring optimal positioning and stability. Then, Le Fort I osteotomy, BSSRO, and genioplasty were performed in accordance with the VSP. All bone segments were fixed, and patients’ occlusion and facial profile were checked. Finally, the oral incisions and conjunctival incisions were tightly sutured (Figure 3).

### 2.5. Evaluation

In our study, we evaluated the postoperative outcomes looking at three aspects: (1) the accuracy of orthognathic and reconstructive surgery with the guidance of customized 3D-printed surgical adjuncts; (2) the orbital and zygomatic symmetry of each patient after surgery; and (3) the resorption rate of the grafted bone six months after surgery.

**Figure 4:**
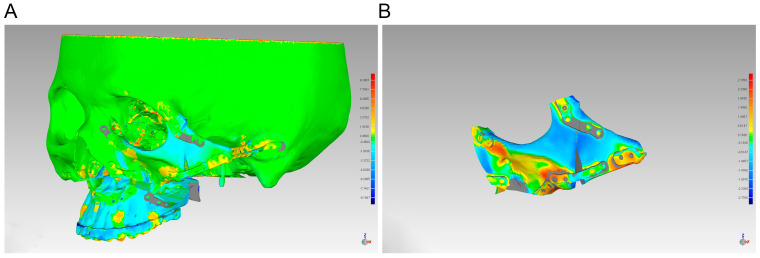
Discrepancy analysis of zygoma’s alignment between simulation and real result. (**A**) Preoperative and postoperative skull models at 3 days were imported into Geomagic Studio 2013 Software for alignment. (**B**) Discrepancy analysis of alignment between preoperative and postoperative zygoma.

The accuracy of the orthognathic and reconstructive surgery was evaluated by comparing the actual outcome with preoperative digital planning. Each patient received a craniomaxillofacial CT scan three days after the surgery. First, the ProPlan CMF software was employed to reconstruct 3D virtual skull models from both preoperative and postoperative CT scans. The processes of superimposition and reference point determination were conducted in the “Scan registration wizard” of the “Segment” module and the “Measure and Analysis” of the “CMF/Simulation” mode in ProPlan CMF software. Skull models from virtual design and actual postoperative outcomes were registered and fused to calculate their differences as an indicator of accuracy. We applied Geomagic Studio 2013 Software to align the actual postoperative zygomas, orbits, and a portion of the maxilla with the virtual planned model, in which a least-mean-squared algorithm was employed to achieve 3D surface-to-surface matching. Mean and maximum 3D deviation were then measured to quantify the deviations (Figure 4).

**Figure 5:**
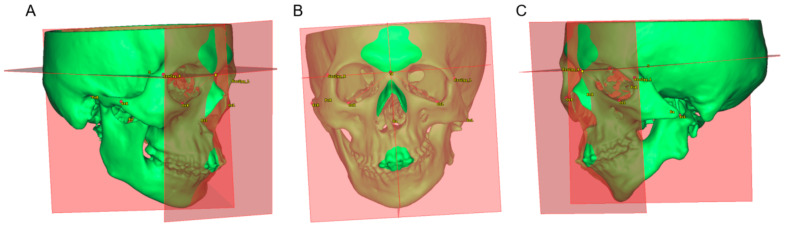
Determination of landmark points and reference planes. The Or, Er, FZ, and three mutually perpendicular reference planes established with the N point as the zero point are shown bilaterally from (**A**) the right 45°, (**B**) anterior view, (**C**) and left 45°, respectively.

To assess the orbital and zygomatic symmetry quantitatively, we marked reference points and compared the positions of anatomical landmarks before and after the surgery. Six fundamental marker points were identified on the virtual skull models: Nasion (N), bilateral Orbitale (OrL, OrR), bilateral Porion (PoL, PoR), Sella (Se), Basion (Ba), and the midpoint of the zygomaticotemporal suture [18] and Fronto-zygomatic suture [19] (ErL/R and FZL/R) (Table 1). Based on the points Ba, Se, and N, the sagittal plane (SP) was determined. In this standardized coordinate system with three orthogonal reference planes (x, y, z), point N was established as the origin, aligning the sagittal plane (SP) with the y-plane (Figure 5). The perpendicular distances from anatomical landmarks to the reference planes (x-plane, y-plane, z-plane) were measured as *dx*, *dy*, and *dz*, respectively. For each paired bilateral landmark, the differences in *dx*, *dy*, and *dz* between the right side (“*R*”) and left side (“*L*”) revealed the three-dimensional differences of paired landmarks. Three paired landmarks, Or, Er, and FZ, were selected to assess orbital and zygomatic symmetry. Based on Or, Er, and FZ, we calculated asymmetry index (*AI*) for each point using the following formula:

$$AI=\sqrt{{\left(Rdx-Ldx\right)}^{2}+{\left(Rdy-Ldy\right)}^{2}{+(Rdz-Ldz)}^{2}}$$

The cancellous bone was harvested from the patient’s anterior superior iliac spine and filled into a titanium mesh scaffold. The volume of grafted bone and the extent of bone absorption are critical factors influencing postoperative facial aesthetics. Consequently, attention should be paid to bone resorption rates in the grafted area. Therefore, we collected and compared preoperative and six-month postoperative CT scans to evaluate the bone absorption. The volume of the harvested graft was quantified as V0, while the volume of the bone graft observed six months postoperatively was quantified as V6. The bone resorption rate (*RR*) was subsequently calculated using the following formula:

$$RR=\frac{V0-V6}{V0}\times 100\%$$

### 2.6. Statistical Analysis

To ensure data reliability, all predefined procedures were executed by two independent evaluators. Each patient’s data underwent two separate rounds of locating and analysis. The final results represent the arithmetic mean of these two independent measurements.

Statistical analysis and visualization were performed in SPSS version 21 (IBM, Chicago, IL, USA) and GraphPad Prism version 8 (GraphPad Software, La Jolla, CA, USA). The Kolmogorov–Smirnov and Shapiro–Wilk tests were employed to assess the normality of the distribution. Data were presented as mean ± standard deviation (SD). Differences were analyzed by paired t test if the variables were normally distributed. If not, the Wilcoxon signed-rank test was used. *p* < 0.05 (*) was considered statistically significant.

## 3. Results

**Figure 6:**
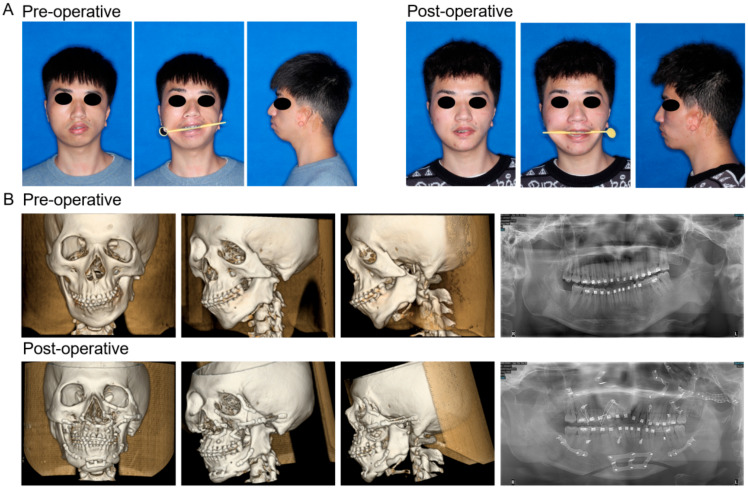
Preoperative and postoperative (6 months later) facial photographs (**A**), CT images, and panoramic radiographs (**B**).

Five patients (two male and three female) were included in this study. Utilizing fully digital treatment plans, all participants achieved satisfactory clinical results (Figure 6A), devoid of severe infections and other complications. Postoperative CT scans were performed on the third day as well as at the 6-month follow-up (Figure 6B). 

Combined with the results of the model alignment, the patient’s postoperative position of the maxilla, zygoma, and orbital rim was consistent with the virtual surgical plan, with only a slight increase in the area of bone grafting (Figure 4). In all enrolled patients, the mean 3D deviation of the actual zygoma segments compared with the virtual plan was 1.07 ± 0.15 mm, while the maximum 3D deviation was 3.11 ± 0.44 mm. The observed bone resorption rate at 6-month follow-up across the five patients was 45.24% ± 3.13% (Table 2).

**Figure 7:**
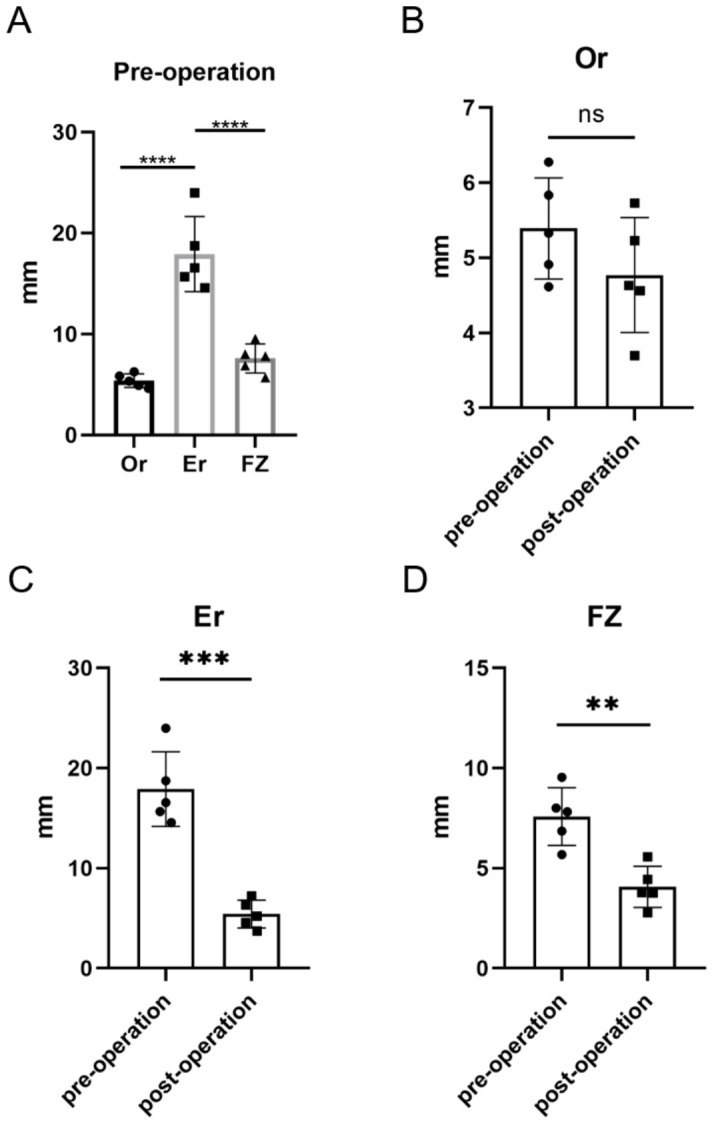
The orbital and zygomatic symmetry assessment. (**A**) Preoperative AI in Or, Er, and FZ and demonstrated preoperative asymmetry of the zygomatic bones in HFM patients was primarily influenced by Er. Comparison of asymmetry index (AI) in (**B**) Or, (**C**) Er, and (**D**) FZ between pre operation and post operation. ** *p* < 0.01, *** *p* < 0.001, **** *p* < 0.0001, ns, no significance.

In terms of the effectiveness of the surgery, the zygomatic and orbital symmetry was significantly improved (Figure 6). Quantitatively, Figure 7A showed that preoperative asymmetry of the zygomatic bones in HFM patients was primarily influenced by Er. The AI of the landmarks Er and FZ significantly decreased (AI of Er: from 17.91 to 5.42 mm, *p* = 0.0001; AI of FZ: from 7.58 to 4.07 mm, *p* = 0.0009). However, the AI of Or was not significantly changed from pre operation (*p* = 0.2089) (Figure 7).

## 4. Discussion

Hemifacial Microsomia is a complex craniofacial disorder, characterized by a spectrum of phenotypes with varying degrees of severity [3]. This condition encompasses skeletal and soft tissue aberrations that pose significant challenges for its treatment. The correction of facial skeletal deformities is paramount given their profound impact on the development of soft and associated non-skeletal tissues [20]. Orthognathic surgery serves as a primary intervention to rectify these skeletal abnormalities in HFM patients, with the goal of enhancing facial symmetry and aesthetics [4,21]. Optimal surgical results are achieved when the delineated preoperative strategy is meticulously implemented and communicated during the surgical intervention.

Predominantly, patients with HFM showcase facial anomalies that are marked by mandibular deviation and abnormal condylar morphology [22,23]. Previous studies have largely focused on the nuances of mandibular deviation, asymmetry, and the postoperative stability of both the condyle and its adjacent articular fossa [24,25,26]. However, there is a relative dearth of research on the midface deformities in HFM patients such as zygomatic arch, maxilla, and orbits, despite their origin from the first pharyngeal arch [27]. Alterations in the position of the zygoma can influence the volume of the orbit, potentially leading to irregular eye movements or, in severe cases, vision impairment or blindness [28]. Thus, the accuracy of the zygomatic osteotomy during surgery and the exact three-dimensional positioning of the bone segments are of paramount importance. To enhance surgical precision, VSPs were performed on all five patients, and 3D-printed personalized titanium osteotomy guides along with fixed titanium plates were designed and fabricated. This ensured that the movement of the bone segments aligned with the desired VSP outcome. Among the five patients included in this current study, the mean 3D deviation between the preoperatively designed zygoma and the actual zygoma at 3 days post operation was 1.07 ± 0.15 mm. Additionally, the maximum 3D deviation was 3.11 ± 0.44 mm. Recently, Si et al. [24] studied the effectiveness of navigation using personalized guides for condylar resection in patients with HFM and found that the average 3D cutting deviation in the guide-assisted osteotomy group was 1.20 ± 0.60 mm, with the maximum deviation being 2.36 ± 0.51 mm. Similarly, Zhu et al. [29] examined the precision of mandibular angle osteotomies and reported an error of 0.96 ± 0.42 mm in the guide group. Comparatively, previous studies assessing VSP accuracy reported mean and maximum 3D deviation results of approximately 1 mm, which closely resemble the findings of the current study. Therefore, it can be confidently concluded that the utilization of 3D-printed titanium cutting guides demonstrates a high level of feasibility in zygomatic reconstruction.

The zygoma, often described as a quadrangular or diamond-shaped irregular formation, constitutes the anterior and lateral aspects of the facial skeleton [30]. It is indispensable to the function of occlusion and plays a vital role in defining facial aesthetics [31,32]. The current study found that postoperative measurements revealed a significant enhancement in the asymmetry indices for both Er and FZ points on the affected side of the patient’s face. However, no significant change was observed in the AI for the Or point. Notably, preoperative comparisons underscored that the asymmetry of the Er was markedly more pronounced compared with the other two focal points, FZ and Or. The possible explanations for these outcomes are twofold. Firstly, all the patients included in this study were categorized as either Pruzansky–Kaban type IIb or III. Moreover, the orbital deformities in the patients are classified as Type O1 within the “OMENS+” classification, exhibiting only minor vertical asymmetries. These classifications indicate a less severe orbital deformity compared with patients diagnosed with type III or other syndromes that involve zygomatic asymmetry [5]. Secondly, the Er point essentially serves as the midpoint of the most protruding section of the zygomatic arch. Hence, any displacement of the zygomatic bone is more readily noticeable at this specific point [5,30,33]. Therefore, we contend that improvements in symmetry at the Er point serve as a more representative indicator of the overall enhancement in zygomatic symmetry. In the reconstruction of the zygomatic arch, iliac cancellous bone was chosen as the primary graft. Firstly, iliac cancellous bone grafts exhibit fewer adverse effects at the donor site compared with other autogenous bone sources, such as ribs. Additionally, they show fewer autoimmune reactions compared with the use of prosthetic materials [34]. Notably, Omara et al. [35] compared the resorption rate of autogenous mineralized pulp matrix (MPM) with cancellous bone particles from the anterior iliac crest for the repair of an alveolar cleft, where the resorption rate in the control group was 48.91%, which was essentially the same as the bone resorption rate in this study. In the current study, we hypothesize that the elevated rate of bone resorption is attributable to the insufficient depth of the titanium mesh, coupled with suboptimal vascularization in the surrounding tissues compared with areas such as the alveolar eminence. These factors may compromise the osteogenic potential of the grafting site. However, given that the primary objective for reconstruction at this site is external restoration, the observed resorption rate was acceptable.

It is essential to acknowledge the inherent limitations of this study, particularly the limited sample size. Additionally, the study did not independently record certain critical clinical parameters, including the duration of surgery and the volume of blood loss. To enhance the robustness and comprehensiveness of the clinical data, future research should emphasize gathering more extensive long-term observational data. This approach will be instrumental in developing a more thorough and detailed understanding of the clinical outcomes. The current study’s constraints, such as the small sample size and the absence of independently documented clinical details, highlight areas for improvement in subsequent research efforts.

## 5. Conclusions

In conclusion, this study explored a novel approach employing a fully digital treatment methodology for maxillofacial reconstruction in HFM patients, which involves simultaneous orthognathic surgery with the application of 3D-printed cutting guides and fixation plates to correct orbital deformities, along with the utilization of 3D-printed personalized titanium plates and a titanium mesh combined with iliac cancellous bone graft to reconstruct the zygomatic arch. Admirably, with the assistance of VSP and 3D printing surgical tools, the surgical outcomes have become more predictable, precise, and effective. This has greatly improved the postoperative midfacial deformities in HFM patients, while also promoting interdisciplinary collaboration among medical professionals and increasing patient engagement. Ultimately, it has facilitated the smooth completion of the treatment process, laying the foundation for subsequent related therapies.

## Figures and Tables

**Table 1:** Anatomical landmarks.

Landmark		Definition
Na	Nasion	Midpoint of the frontonasal suture
Po *	Porion	Most superior point of the external acoustic meatus
Or *	Orbitale	Most inferior point of the infraorbital rim
FZ *	Frontozygomatic suture	Midpoint point of the frontozygomatic suture at the level of the lateral orbital rim
Er *	Zygomaticotemporal suture	Midpoint of the zygomaticotemporal suture
Se	Sella	Midpoint of the pituitary fossa
Ba	Basion	The most inferior point on the anterior margin of the foramen magnum in the middle

* indicates that these points are bilateral landmarks.

**Table 2:** Accuracy of postoperative outcomes of zygomatic reconstruction compared with virtual surgical planning.

Sample	Gender	Age (years)	Mean 3D Deviation (mm)	Max 3D Deviation (mm)	Bone Resorption Rates
1	F	24	0.98	2.84	40.64%
2	F	19	1.00	2.87	45.29%
3	F	21	1.33	3.82	43.28%
4	M	26	0.97	3.26	49.73%
5	M	24	1.05	2.77	47.24%
Mean ± SD		22.8 ± 2.77	1.07 ± 0.15	3.11 ± 0.44	45.24% ± 3.13%

## Data Availability

All data generated and/or analyzed during the current study are available from the corresponding author on reasonable request.
